# Influence mechanism exploration and machine learning prediction of loess compression deformation coefficient under multi-factor coupling effects

**DOI:** 10.1371/journal.pone.0338428

**Published:** 2026-01-09

**Authors:** Wei Zhou, Changqing Deng, Jin Wang

**Affiliations:** 1 College of Civil and Architectural Engineering, Shaoyang University, Shaoyang, Hunan, China; 2 Key Laboratory of Green Construction and Intelligent Monitoring in Southwestern Hunan for Regular Institutions of Higher Education of Hunan Province, Shaoyang, Hunan, China; 3 Hunan Engineering Research Center for Bamboo Fiber Construction Materials, Shaoyang, Hunan, China; Henan Polytechnic University, CHINA

## Abstract

Accurate prediction of the compression deformation coefficient of loess fillers is key for stability assessment of loess subgrade engineering. In this study, the effects of key influencing factors such as compaction, water content, vertical pressure and molding method on compression characteristics were investigated. Four machine learning models, XGBoost (XGB), Support Vector Regression (SVR), Backpropagation Neural Network (BP), and Sparrow Search Algorithm-optimized BP (SSA-BP), were developed to predict the deformation coefficient using experimental datasets. SHAP interpretability analysis quantified feature contributions and coupling effects. Results demonstrate that: Vibration compaction, increased compaction, and reduced water content enhance particle interlocking, thereby effectively suppressing deformation and reducing the compression deformation coefficient; The coefficient significantly increases with rising vertical pressure in compacted loess. The metaheuristic-optimized SSA-BP model demonstrated superior performance with a test set RMSE of 0.138%, significantly outperforming both the SVR、XGB and standard BP models by 35%、45%and 46%, respectively. SHAP analysis revealed vertical pressure as the most influential factor and identified significant nonlinear interactions, particularly between vertical pressure and water content. These findings provide both a reliable prediction tool and mechanistic insights for loess subgrade engineering.

## 1. Introduction

Loess, as a unique Quaternary sediment widely distributed in mid-latitude arid and semi-arid regions, covers approximately 10% of the global land area and serves as the most commonly used subgrade filler in road engineering [1–4]. Its distinctive metastable structure, characterized by high porosity and sensitivity to water, makes it prone to compression deformation and collapsibility under hydro-mechanical coupling effects. The compression deformation characteristics of compacted loess fillers directly govern subgrade settlement evolution and long-term operational safety [5–10]. Therefore, identifying these characteristics, analyzing the influencing factors, and developing reliable prediction models are paramount for ensuring the stability and durability of infrastructure built on loess substrates.

Accurately predicting the compression deformation coefficient of compacted loess is crucial for subgrade design and safety assessment. However, achieving this is challenging because compression deformation coefficient is simultaneously governed by the complex coupling of multiple factors, including compaction energy, moisture condition, stress level, and construction method. Traditional empirical or theoretical models often fail to fully capture these intricate, nonlinear interactions. Meanwhile, while machine learning (ML) shows promise, its application in loess mechanics has predominantly focused on collapsibility, leaving a gap in the prediction of the fundamental compression deformation coefficient under multifactorial coupling effects.

This study integrates laboratory experimentation with advanced machine learning techniques to explore the mechanisms and prediction of the loess compression deformation characteristic. The primary objectives are: (1) To investigate the individual and coupled effects of compaction, water content, vertical pressure, and molding method. (2) To develop and compare the performance of four machine learning models—XGBoost (XGB), Support Vector Regression (SVR), Backpropagation Neural Network (BP), and Sparrow Search Algorithm-optimized BP (SSA-BP)—for accurately predicting the compression deformation coefficient. (3) To employ SHAP interpretability analysis on the optimal model to quantify feature importance and reveal interaction effects.

The rest of this paper is organized as follows. Section 2 (Related Work) provides a detailed review of previous studies on loess compression behavior and machine learning applications, critically analyzing their contributions and limitations to further clarify the positioning of this research. Section 3 (Experimental and Methodology) describes the materials, specimen preparation methods, testing procedures, and the acquisition of the dataset. Section 4 (Analysis of Factors Affecting) presents a detailed analysis of the individual effects of compaction, vertical pressure, water content, and molding method on the compression deformation coefficient. Section 5 (Machine Learning-based Compression Performance Prediction) elaborates on the construction, evaluation, and comparison of the four machine learning models, and employs SHAP analysis for model interpretation. Finally, Section 6 concludes the study by summarizing the main findings and outlining potential directions for future research.

## 2. Related work

A substantial body of research has been dedicated to understanding and predicting the compression behavior of loess. Traditional approaches have primarily relied on laboratory experiments and theoretical models. Experimentally, scholars have investigated the impact of various factors. Jiang et al. demonstrated that vibration-compacted specimens exhibit superior deformation resistance compared to static compaction, establishing the importance of molding method on initial soil fabric [11]. Leng et al. developed a constitutive model for loess collapsibility through triaxial tests, providing valuable insights into water-induced deformation mechanisms, though their model remains specific to collapse behavior under particular stress paths [12]. Hao et al. emphasized that matrix suction and net confining pressure jointly regulate deformation characteristics of unsaturated compacted loess, advancing understanding of hydro-mechanical coupling [13]. Cai et al. discovered that wetting-induced deformation under unloading paths exceeds that under loading paths, highlighting the significance of stress history [14], while Chai et al. confirmed that increased isotropic stress exacerbates compression deformation [15]. These stress-path studies, however, were conducted under fixed initial conditions, limiting their applicability across varying compaction states and water contents. Hu et al. developed a permanent strain prediction model through cyclic loading tests, revealing cumulative deformation mechanisms [16], and Hu et al. optimized loess compaction techniques [17]. While these studies provide valuable practical insights, their models are specific to particular loading conditions (dynamic vs. static) and cannot be directly applied for general compression deformation prediction. Collectively, these traditional approaches successfully identify individual factor effects but fail to characterize nonlinear interactions among multiple factors, as their methodologies are inherently limited by the reductionist approach of isolating variables [18–23]. There is a scarcity of experimental studies that investigate the multi-factor coupling effects on the loess compression deformation behavior. Existing research often focuses on the influence of individual factors.

In recent years, data-driven machine learning (ML) methods have emerged as powerful tools for capturing complex nonlinear relationships in geotechnical engineering [24–27]. These approaches can efficiently establish mappings between input parameters and material responses from extensive datasets, reducing reliance on idealized assumptions and potentially offering higher accuracy [28–31]. While ML has been successfully applied to various subgrade performance prediction problems, most existing applications in loess research have focused primarily on collapsibility assessment. Specifically, Mu et al. employed multiple expression programming (MEP) and backpropagation neural network (BPNN) to evaluate loess collapse susceptibility, making a significant contribution by introducing a hybrid modeling approach that combines the advantages of explicit mathematical expressions from MEP with the powerful nonlinear mapping capability of BPNN, thereby providing both accurate predictions and interpretable functional relationships for collapse assessment [32]. Zhang et al. demonstrated that Bayesian-optimized random forest models can reliably predict the collapsibility coefficient of loess, with their main contribution lying in the innovative integration of Bayesian optimization for automatic hyperparameter tuning, which substantially enhances model generalization ability [33]. Sahand et al. compared three supervised machine learning tools for estimating loess collapse potential, with they evaluates different algorithm families and identifies the Multi-Layer Perceptron Neural Network as the most accurate model, thereby providing valuable guidance for researchers and practitioners in selecting appropriate modeling techniques for collapsibility prediction [34]. However, the application of ML specifically to predicting the compression deformation coefficient of loess—a crucial parameter for settlement calculation in subgrade engineering—remains relatively limited, particularly in addressing the coupled effects of compaction, water content, vertical pressure, and molding method. Furthermore, the application of data-driven machine learning for predicting the loess compression deformation coefficient remains largely uninvestigated.

## 3. Experimental and methodology

### 3.1. Testing and dataset acquisition

#### 3.1.1. Materials.

The test soil samples were collected from an railway project, classified as Q3 aeolian loess (late Pleistocene age). The soil appears brownish-yellow and consists predominantly of clay particles with silt as the secondary component. It exhibits uniform texture and is categorized as low liquid limit silty clay. Through heavy compaction tests, the optimal water content of the loess was determined to be 12%, with a maximum dry density of 1.894 g/cm³. Key physical properties are listed in Table 1, and particle size distribution is detailed in Table 2.

**Table 1:** Main physical property indexes of loess.

Indicator	Specific gravity (g/cm^3^)	Liquid limit (%)	Plastic limit (%)	Plasticity index (%)
Measured value	2.74	36	21.5	14.5

**Table 2:** Particle size distribution of loess.

Particle size (mm)	≤0.005	0.005 ~ 0.05	0.05 ~ 0.075	0.075 ~ 0.25
Mass ratio (%)	25.74	65.94	5.79	2.53

#### 3.1.2. Specimen preparation method.

All specimens were cylindrical with dimensions of diameter × height = 100 mm × 100 mm. Before molding the specimens, air-dried loess samples were evenly spread on a metal plate. The calculated amount of water was uniformly sprayed onto the samples, followed by thorough mixing using stirring tools until homogeneous. Specimens were prepared using two methods: hydrostatic method and vibration method. The hydrostatic method and vibration method refer to the techniques of compacting loess filler to a specified compaction degree and forming samples of specified dimensions using a pressure press and a vibratory compactor, respectively. For the hydrostatic method, the loading rate was controlled at 1 mm/min until the desired specimen height was achieved, after which the pressure was maintained for 2 minutes. For the vibration method, the operational parameters were set as follows: a working frequency of 35 Hz, a counterweight of 300 kg, and a vibration duration of 60 s.

After preparation, specimens were wrapped in plastic film and cured in a standard maintenance chamber at 20 ± 2°C with >95% relative humidity for 48 hours to ensure moisture homogenization prior to testing.

#### 3.1.3. Test method.

**Fig 1:**
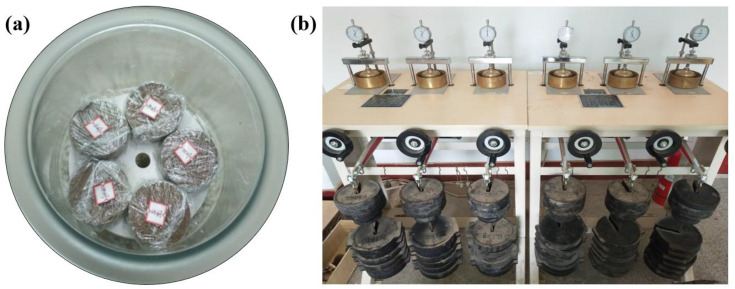
Test specimens and triple-medium pressure consolidometer.

Prior to testing, compression test specimens were extracted from the loess samples using a cutting ring with dimensions Φ61.8 mm × h20 mm (Fig 1a). Specimens were placed in a triple-medium pressure consolidometer and subjected to graded loading. The next vertical pressure level was applied only when the deformation rate under the current pressure stabilized below 0.01 mm/h (Fig 1b). The selected vertical pressures were 50, 100, 200, 400, 800, and 1600 kPa.

Compression deformation coefficient (*δ*_c_) was calculated using Equation (1), referenced from the definition of collapsibility deformation coefficient [35]:


$$\delta_{c}=\frac{h_{\text{0}}-h_{\text{1}}}{h_{\text{0}}}$$ (1)

where: *h*_0_ is the initial height of the specimen; *h*_1_ is the height of the specimen after deformation stabilization under pressure.

#### 3.1.4. Dataset acquisition.

**Fig 2:**
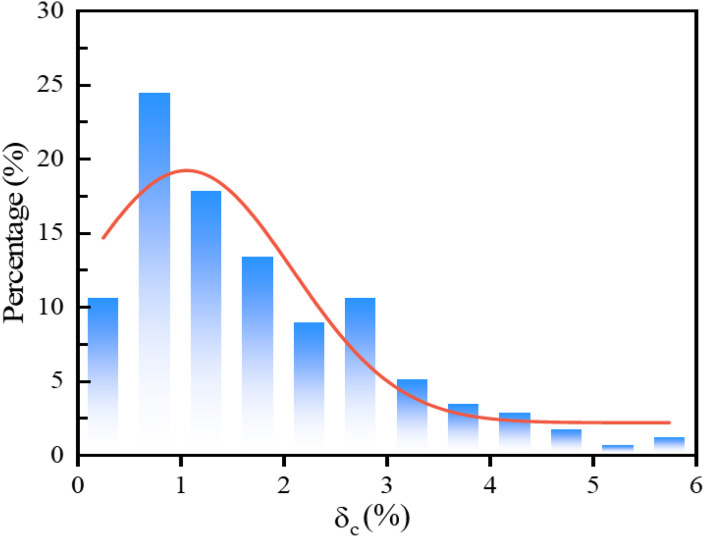
Results of data distribution.

A experimental program was designed to investigate the effects of key factors on the compression deformation coefficient. Specimens were prepared using both vibration and hydrostatic methods, with water content (12%, 14%, 16%), compaction degree (0.90, 0.93, 0.96, 0.98, 1.00), and vertical pressure (50, 100, 200, 400, 800, 1600 kPa) as the controlled variables. This full-factorial design resulted in a robust dataset of 180 data points, with each point representing the average of three parallel experiments to ensure reliability. Each data point represents the average value of three parallel experiments after excluding any outliers identified through the Grubbs’ test. The standard deviation of the triplicate measurements for all test conditions was controlled to be less than 0.05%, ensuring repeatability and reliability. The resulting dataset, partially displayed in Table 3, captures the compression deformation coefficient across a wide range of conditions. It is notable that the complete database is shown in S1 Table. The distribution of the entire dataset is visualized in Fig 2, confirming its suitability for subsequent machine learning analysis.

**Table 3:** Partial sample data.

Number	Molding method	Vertical pressure (kPa)	Water content (%)	Compaction	*δ*_c_ (%)
1	Hydrostatic method	50	12	0.9	0.45
2	Hydrostatic method	100	12	0.9	0.8
3	Vibration method	50	12	0.96	0.31
4	Vibration method	50	16	0.96	0.53
5	Hydrostatic method	800	14	0.98	2.13
6	Vibration method	800	12	0.96	1.82
7	Hydrostatic method	1600	12	0.98	2.74
8	Vibration method	1600	14	1	2.76
9	Hydrostatic method	50	16	0.98	0.48
10	Vibration method	100	12	0.9	0.79
…	…		…	…	…

### 3.2. Machine learning model construction

#### 3.2.1. Machine learning algorithms.

(1) **Extreme Gradient Boosting (XGB)**

**Fig 3:**
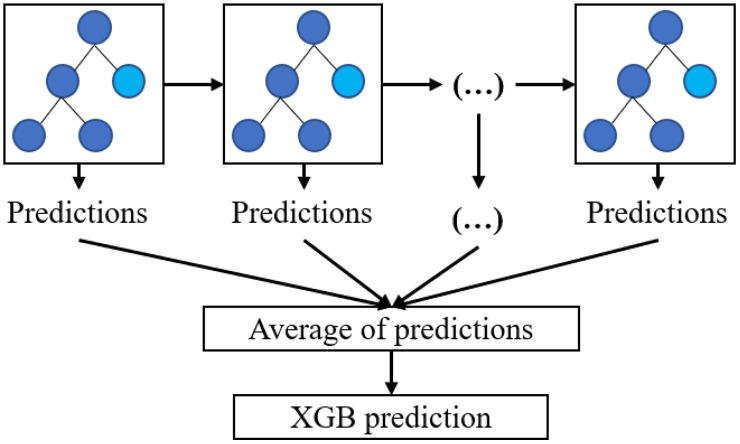
Schematic diagram of XGB algorithm.


XGB is a scalable tree ensemble framework that significantly enhances gradient boosting through regularized objective function optimization, the schematic is shown in Fig 3. By employing second-order Taylor expansion for precise loss function approximation and incorporating regularization parameters to penalize model complexity, it effectively balances accuracy and overfitting control [36]. The objective function of the XGB model is shown in Eq. (2).

$$\begin{array}{c} F=\sum {\mathrm{\backslash nolimits}}_{i=\text{1}}^{n}L(y_{i},{{\hat{y}}_{i}}^{\left(t-\text{1}\right)}+f_{t}\left(x_{i}\right))+\Omega (f_{t}) \end{array}$$ (2)

Where *L*, *x*_*i*_, Ωare the mass loss, the *i* th input variable, the regularization term, respectively.

(2) **Support Vector Regression (SVR)**

**Fig 4:**
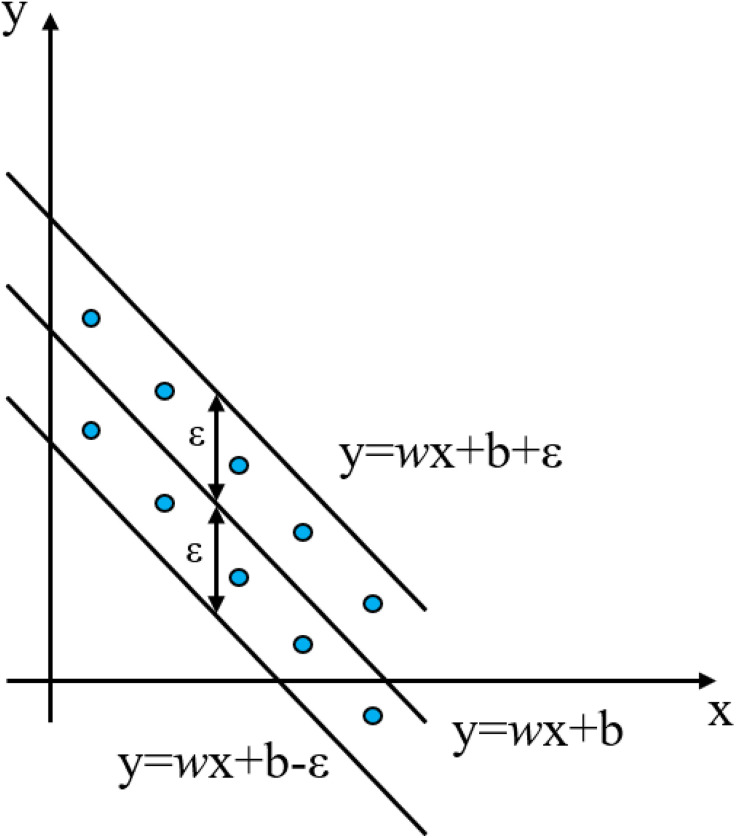
Schematic diagram of SVR algorithm.


SVR is a powerful supervised learning algorithm derived from Support Vector Machines, designed specifically for regression tasks. Its core objective is to find a function that approximates the continuous target variable while maintaining a balance between model complexity and fitting error. A key feature is the ε-insensitive loss function, which creates a tube of width ε around the predicted function. Errors within this tube are ignored, focusing optimization only on points lying outside it, as depicted in Fig 4. SVR minimizes a regularized loss function to prevent overfitting and utilizes kernel functions to implicitly map input data into high-dimensional feature spaces, enabling effective modeling of complex nonlinear relationships [37,38]. The objective function of the SVR model is shown in Eq. (3).

$$\begin{array}{c} F=\text{min}\frac{\text{1}}{\text{2}}{\left\|\omega \right\|}^{\text{2}}+C\sum {\mathrm{\backslash nolimits}}_{i=\text{1}}^{n}l_{\varepsilon }(z^{\left(i\right)}-z^{\left(i\right)}(\omega^{T}f(x{)}^{\left(i\right)}+d)) \end{array}$$ (3)

Where *w*, *d*, *C*, *n*, *l*ε, *z*, *x* are the regression coefficient, the bias term, the penalty coefficient, the number of samples, the insensitivity coefficient reflecting the error tolerance area, the actual value, and the input variable, respectively.

(3) **Back Propagation (BP)**

**Fig 5:**
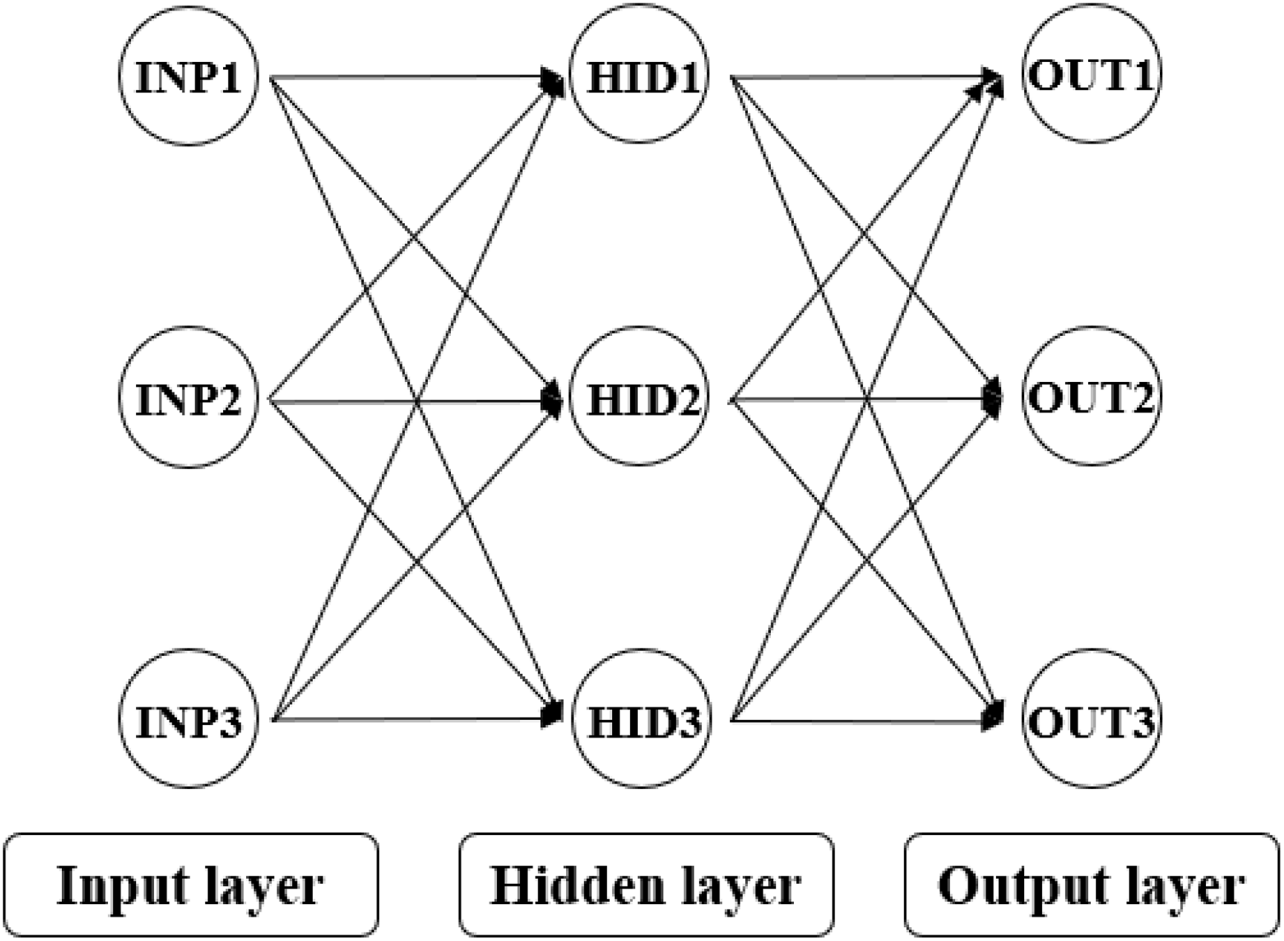
Schematic diagram of BP algorithm.

Backpropagation (BP) neural networks constitute a foundational supervised learning architecture that implements gradient-based optimization through multilayer feedforward structures [39]. The algorithm operates in two critical phases: Forward propagation computes outputs via weighted summations and nonlinear activation functions, while backward propagation dynamically adjusts synaptic weights by calculating the gradient of the loss function with respect to network parameters using the chain rule [40]. This iterative error-minimization process enables BP to approximate complex nonlinear mappings through distributed hierarchical representations. The schematic diagram of BP algorithm is shown in Fig 5.

(4) **Sparrow Search Algorithm Optimized Backpropagation (SSA-BP)**

**Fig 6:**
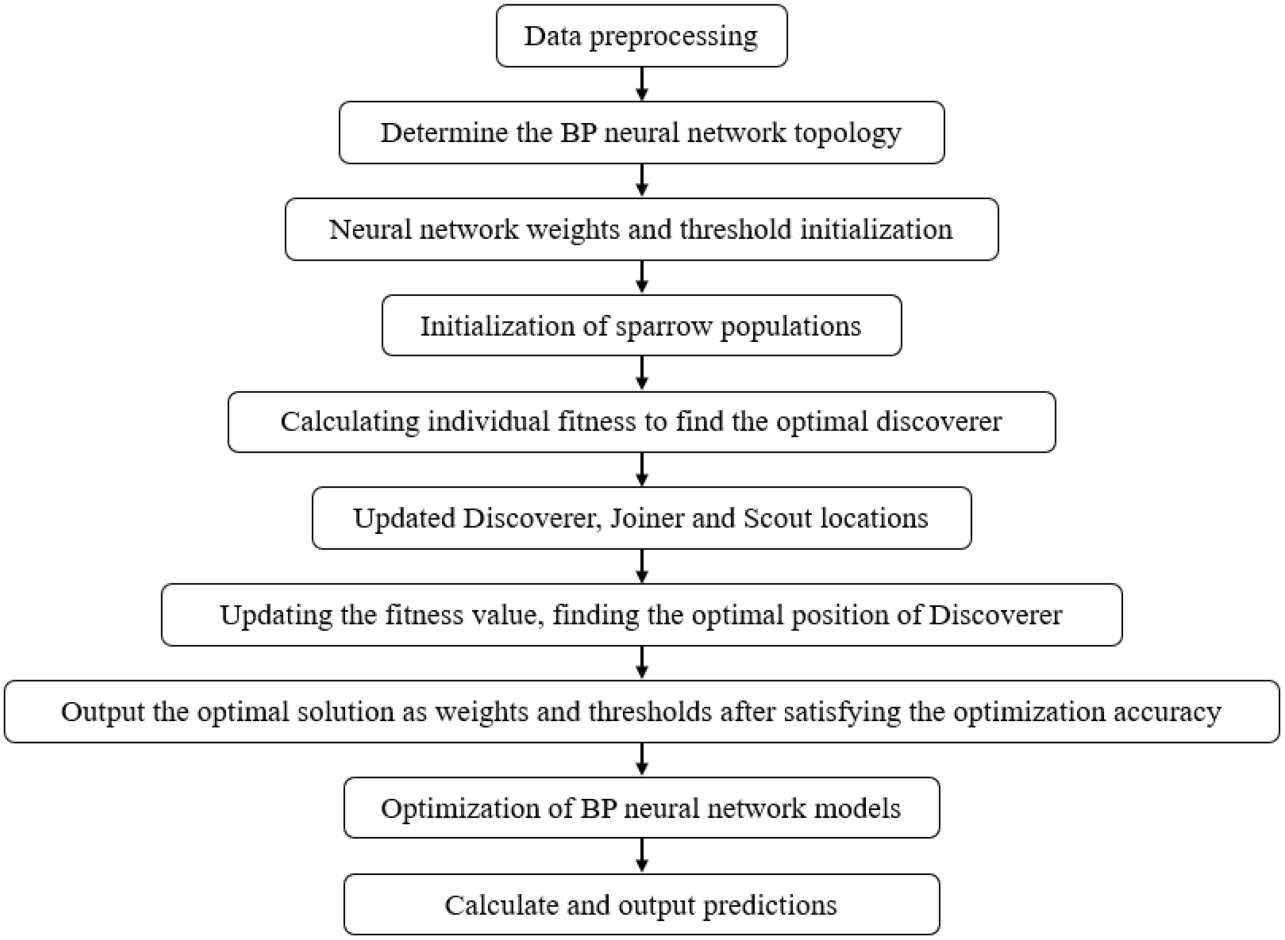
Flowchart of SSA-BP algorithm.

SSA-BP is a hybrid intelligent model that integrates the global search capability of the bio-inspired Sparrow Search Algorithm (SSA) with the local optimization strength of Backpropagation (BP) neural networks. SSA first optimizes the initial weights and thresholds of the BP network by simulating sparrow foraging and anti-predation behaviors, effectively escaping local optima and accelerating convergence. The refined parameters are then transferred to the BP network for precise gradient-based fine-tuning. This dual-phase optimization mechanism significantly enhances prediction accuracy, generalization ability, and training stability while suppressing overfitting [41]. The construction process of SSA-BP is illustrated in Fig 6.

#### 3.2.2 Model construction.

**Fig 7:**
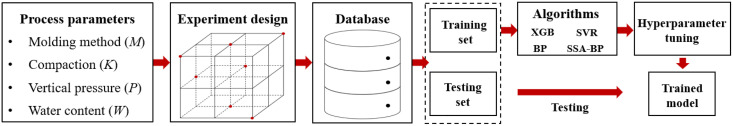
The construction process of the prediction model.

All datasets are uniformly divided into training and test sets in the ratio of 7:3. The training set is used for learning and optimizing the model parameters, while the test set is used for independently evaluating the generalization performance of the model. The construction process of the prediction model is depicted in Fig 7.

**Fig 8:**
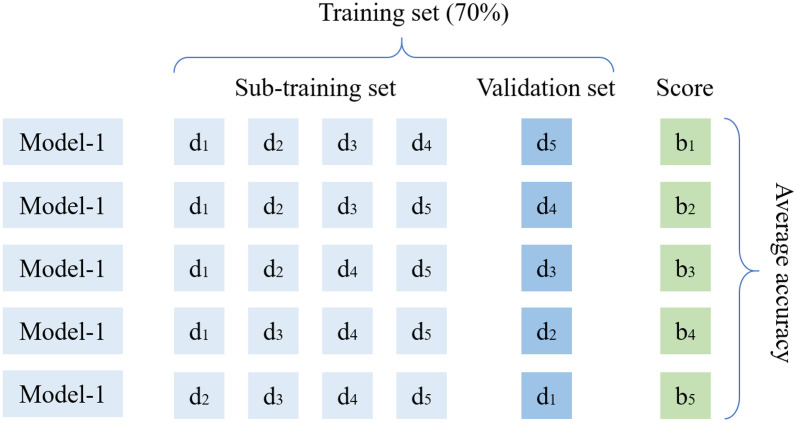
Schematic diagrams of 5-fold cross validation.

Given the limited dataset size (n = 180) from a machine learning perspective, to ensure each model achieves its optimal predictive performance, a comprehensive hyperparameter optimization was performed using grid search combined with 5-fold cross-validation [42]. During the process, the training set was further divided into five equal subsets, with four subsets used for training and the remaining one for validation in each iteration. This cross-validation procedure was repeated five times, with each subset serving as the validation set once. The final performance metric was calculated as the average across all five folds, thereby reducing the risk of overfitting and ensuring the selection of robust hyperparameter configurations, as depicted in Fig 8. Table 4 presents a summary of the optimal hyperparameter configurations for the four models on the dataset.

**Table 4:** Summary of the optimal hyperparameter configurations for the four models.

Algorithms	Hyperparameters	Optimum value
XGB	learning_rate	0.05
	max_depth	6
	subsample	0.8
	gamma	0.1
	lambda	1.0
SVR	C	10.0
	epsilon	0.01
	kernel	RBF
	gamma	0.1
BP	hidden_layers	10
	learning_rate	0.01
	activation	ReLU
SSA-BP	SSA_population	50
	SSA_iter	100

## 4. Analysis of factors affecting

### 4.1. Effect of compaction

**Fig 9:**
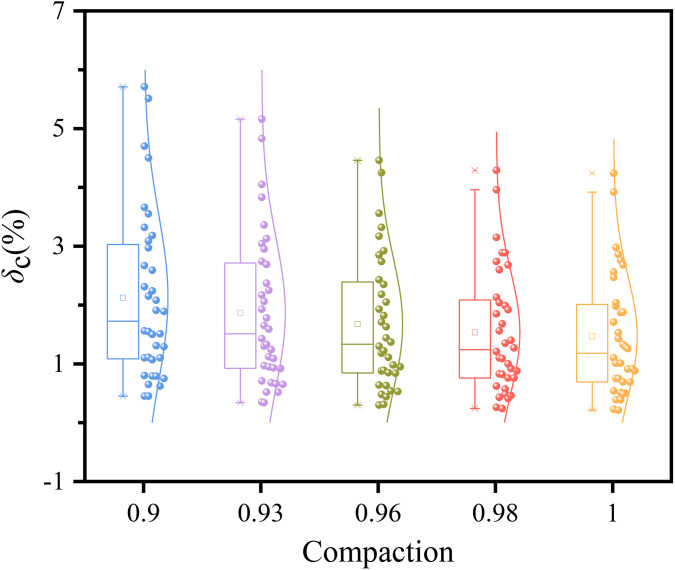
Effect of compaction on the compression deformation coefficient.

Fig 9 illustrates the relationship between the compression deformation coefficient and compaction. Within the dataset, the compression deformation coefficient at identical compaction exhibits a left-skewed distribution, indicating that the majority of samples have coefficients concentrated at lower values. The coefficient decreases progressively with increasing compaction, demonstrating that higher compaction effectively enhances the soil’s resistance to vertical deformation and reduces its compressibility. This occurs because increased compaction leads to tighter particle packing, reduced void ratio, and a more stable structure, resulting in significantly less compression deformation under identical vertical pressure [11].

**Fig 10:**
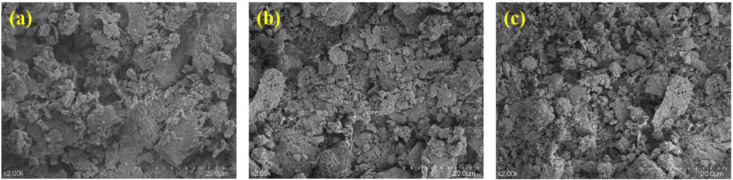
Microscopic morphology of loess under different compaction: (a) K = 0.9; (b) K = 0.96; (c) K = 1.0.

The change of loess micro-morphology with compaction is shown in Fig 10. With the increase of compaction degree, the particle arrangement changed from loose to compact. When the compaction degree is 0.9, the particles are mostly in point contact, and there are a large number of shelf pores and large-diameter connecting pores with irregular pore shapes. When the compaction degree is 0.96, the particles are reorganized by sliding, the point-surface contact is increased, the large pores are divided into small and medium-sized pores by extrusion, and the structural homogeneity is improved. At compaction degree 1.0, the particles are dominated by face contact, the clay minerals are arranged in a significant orientation, the pores are dominated by tiny pores and slit-like pores, and a dense skeleton structure is formed.

### 4.2. Effect of vertical pressure

**Fig 11:**
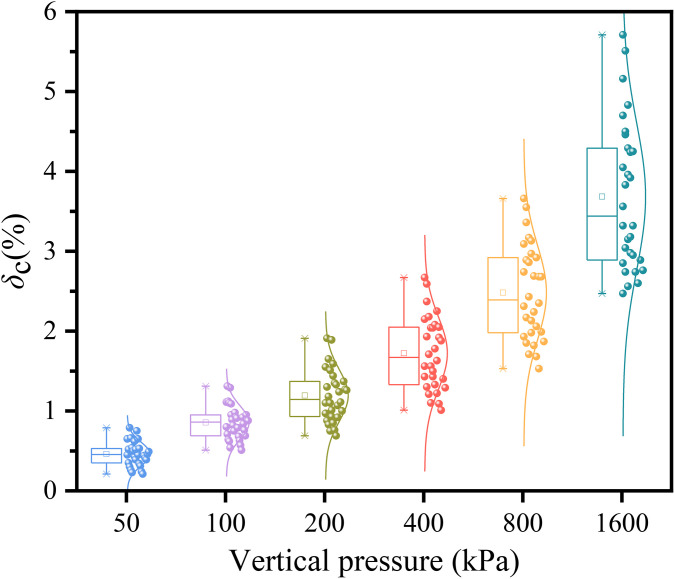
Effect of vertical pressure on the compression deformation coefficient.

Fig 11 depicts the variation of the compression deformation coefficient with vertical pressure. The coefficient shows a significant non-linear increase as vertical pressure rises, but its growth rate gradually diminishes with increasing pressure. This reveals the typical staged nature of soil compression: during the initial stage, rapid compaction of soil pores causes a substantial increase in the deformation coefficient; at higher stress levels, the soil skeleton stabilizes, and the remaining compressible pores decrease markedly, leading to a reduced deformation increment per unit pressure increase. Mechanistically, loose pores are easily compressed under low stress, whereas at high stress, particle rearrangement completes, and the densified structure’s deformation resistance strengthens, causing deformation to approach a limiting value.

**Fig 12:**
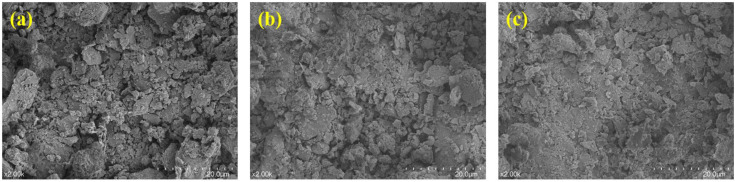
Microscopic morphology of loess under different vertical pressures: (a) P = 0kPa; (b) P = 400kPa; (c) P = 1600kPa.

The change of loess micro-morphology with vertical pressure is shown in Fig 12. When there is no load, the particles keep the initial accumulation and the pore space distribution is loose. With the increase of vertical pressure, the particles slipped and rotated, the large pores collapsed and reorganized into small and medium-sized pores, the contact between particles transitioned from point contact to surface contact, and the viscous particles wrapped the coarse particles to form the “encapsulation structure”. High pressure leads to significant particle fragmentation, broken fines fill the pore space, newborn microcracks develop along the edge of the clay mass, and the structure shows significant directionality.

### 4.3. Effect of water content

**Fig 13:**
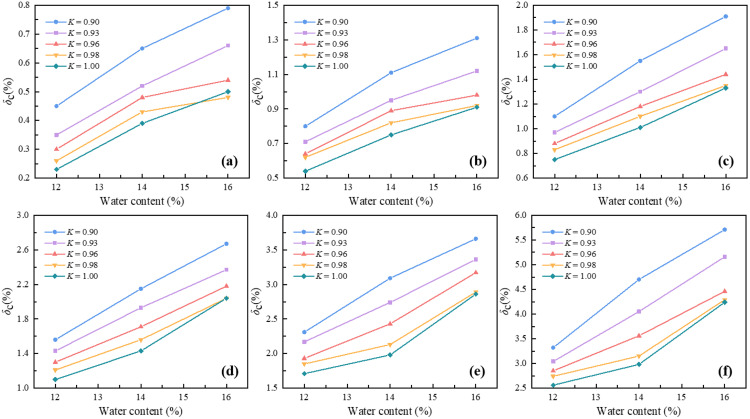
Effect of water content on the compression deformation coefficient: (a) P = 50kPa; (b) P = 100kPa; (c) P = 200kPa; (d) P = 400kPa; (e) P = 800kPa; (f) P = 1600kPa.

Using data from hydrostatic compacted samples (Fig 13), the relationship between the compression deformation coefficient and water content was studied. The coefficient monotonically increases with rising water content, indicating that higher water weakens the soil’s deformation resistance. This positive correlation stems primarily from the lubricating effect of water: increased water reduces inter-particle friction, facilitating particle sliding and rearrangement. Furthermore, considering the soil’s permeability characteristics [43], higher water content enhances hydraulic connectivity between pores, potentially accelerating moisture redistribution during loading. This improved hydraulic conductivity facilitates more uniform pore pressure development, further weakening the soil skeleton. Although fluctuations in the growth rate were observed, their small magnitude may reflect experimental error or local sample heterogeneity.

**Fig 14:**
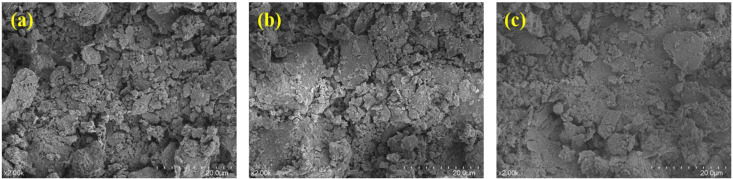
Microscopic morphology of loess under different water contents: (a) W = 12%; (b) W = 14%; (c) W  = 16%.

The variation of loess micro-morphology with water content is shown in Fig 14. At low water content, loess particle contact is dominated by rigid point contact and cementation, and there is more compressive deformation due to void collapse when subjected to compression, whereas with increasing water content, particle contact is dominated by water film connection and plastic surface contact, and thus this deformation tends to be continuous and plastic with a larger total amount when subjected to compressive deformation.

### 4.4. Effect of molding method

**Fig 15:**
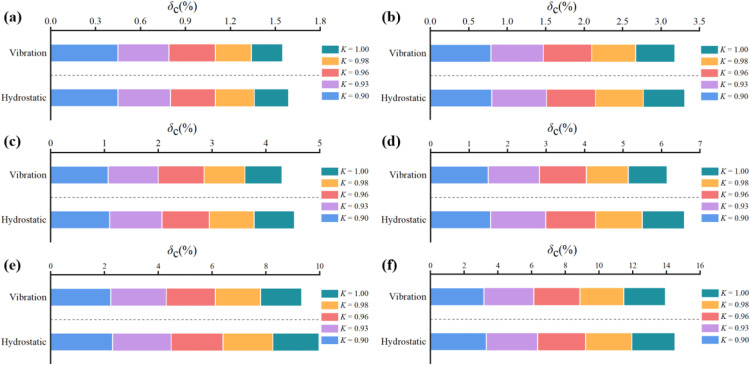
Effect of molding method on the compression deformation coefficient.

Using the dataset of samples with 12% water content as an example, Fig 15 illustrates the variation pattern of the compression deformation coefficient with compaction. The compression deformation coefficient of vibration-compacted loess specimens consistently remains lower than that of hydrostatic compacted specimens, with this difference becoming more pronounced at higher compaction. Quantitative analysis confirms this trend: at low compaction (K = 0.90), the vibration method reduces the compression coefficient by 0–4.2% compared to the hydrostatic method, while at high compaction (K = 1.0), this reduction range expands to 3.5–10.5%. This pattern highlights the superiority of vibration compaction: vibratory energy promotes directional particle rearrangement, forming a more uniform and dense initial structure that enhances deformation resistance. In contrast, hydrostatic compaction relies on static pressure and tends to retain localized pores or weak contact surfaces. As compaction increases, vibration-compacted specimens develop tighter particle interlocking and reduced pore connectivity, strengthening deformation resistance. Conversely, even with higher compaction, hydrostatic compacted specimens remain prone to instability-induced deformation under high stress due to inherent structural defects, thereby widening the performance gap.

**Fig 16:**
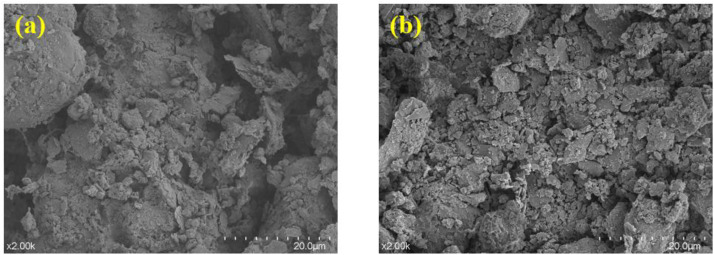
Microscopic morphology of loess under different molding method: (a) Hydrostatic method; (b) Vibration method.

The variation of loess micro-morphology with molding method is shown in Fig 16. Hydrostatic specimens are prone to uneven compaction of the particles, with some areas being dense and others having large voids. The vibration method realizes three-dimensional uniform compaction, the particle contact is dominated by multidirectional point-face contact, the pore shape is nearly isometric, the pore distribution is uniform, and the filling of fine particles between coarse particles is more adequate, but the high-frequency vibration is easy to lead to the crushing of the surface particles.

## 5. Machine learning-based compression performance prediction

### 5.1. Model evaluations

**Fig 17:**
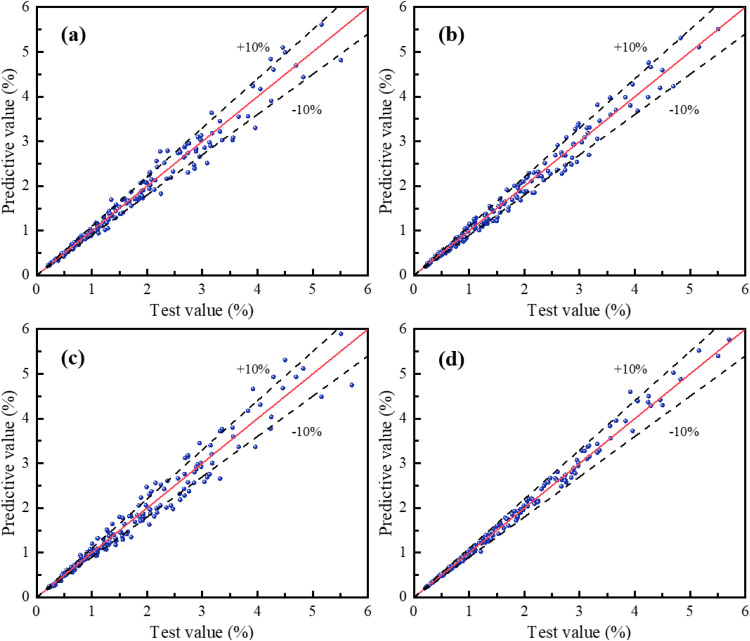
The correlation between test and predictive values for different models: (a) XGB; (b) SVR; (c) BP; (d) SSA-BP.

Fig 17 clearly illustrates the prediction performance of each model through scatter plots comparing test values versus predicted values of the loess compression deformation coefficient for the four algorithm models. Each plot includes a solid diagonal line representing 100% prediction accuracy and two dashed lines (with slopes of 1.1 and 0.9, respectively) defining the ± 10% prediction error bounds. The results reveal that scatter points for both SVR and SSA-BP are more densely clustered between the two error-bound lines and along both sides of the diagonal, indicating their superior prediction performance.

**Fig 18:**
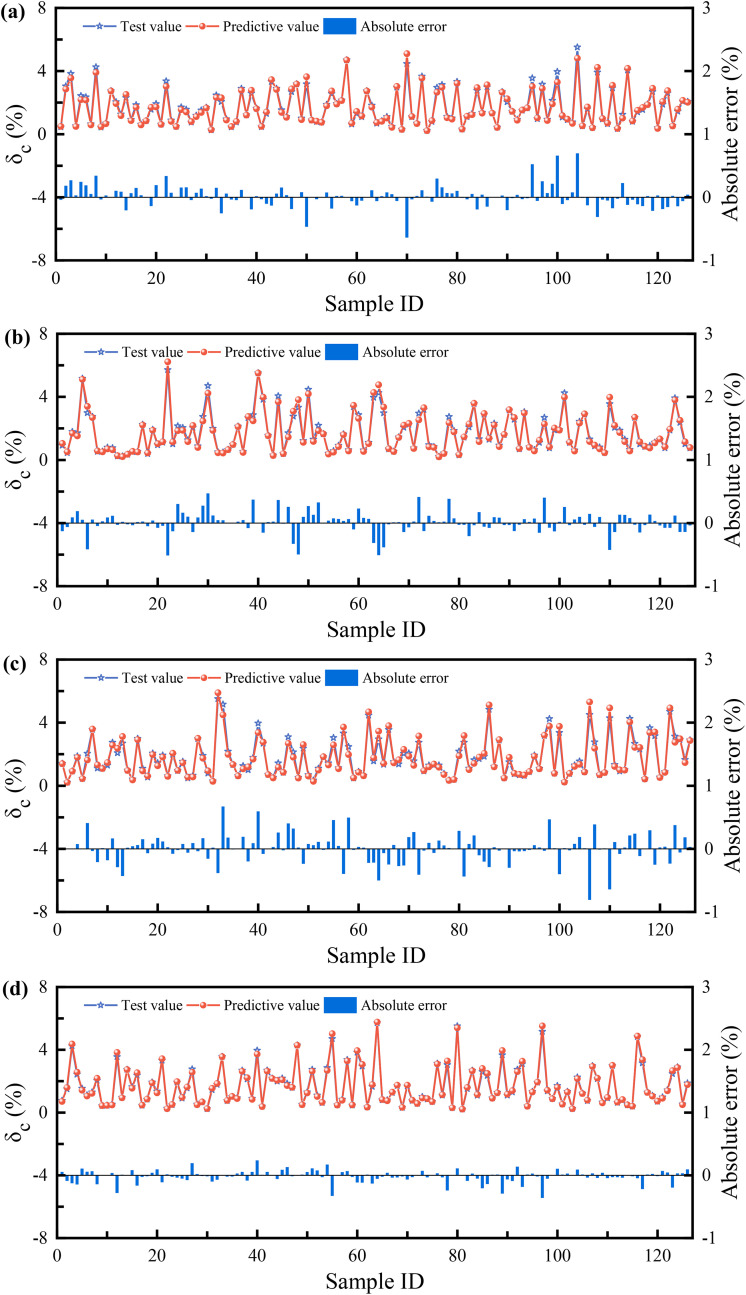
Comparison results of test and predictive values for the training sets: (a) XGB; (b) SVR; (c) BP; (d) SSA-BP.

**Fig 19:**
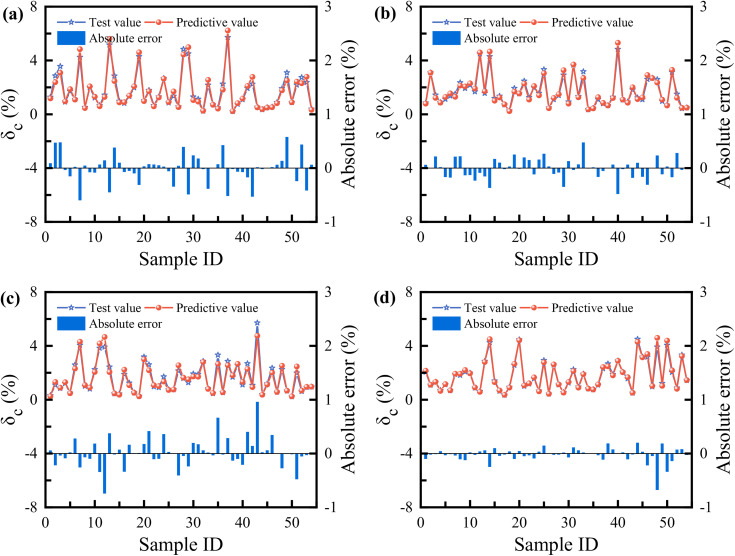
Comparison results of test and predictive values for the testing sets: (a) XGB; (b) SVR; (c) BP; (d) SSA-BP.

Fig 18 clearly demonstrates the performance of each model on the training set, providing a direct visual comparison between the experimentally observed values and the model-predicted values of the loess compression deformation coefficient. Fig 19 further contrasts the generalization capabilities of the models on an independent testing set. A comprehensive analysis integrating absolute error data reveals significant differences in prediction accuracy among the four models (XGB, SVR, BP, SSA-BP).

Specifically, the SSA-BP model delivers the most outstanding performance. Its prediction curve almost completely overlaps with the experimental value curve, demonstrating exceptional fitting and predictive capabilities for both training data and unseen test data. The calculated absolute error values are significantly lower than those of other models, confirming its high precision and stability. The XGB and SVR models exhibit secondary performance. While their prediction curves generally follow the trend of the experimental values, noticeable deviations occur at some extreme-value data points. Their absolute errors exceed those of SSA-BP but remain lower than the BP model, placing their predictions within an acceptable accuracy range. In contrast, the BP neural network model shows the weakest predictive performance. Its prediction curve deviates most significantly from the experimental values across both training and testing sets, particularly near regions of complex data variation or extreme points. The higher absolute error values indicate relatively inferior prediction accuracy and generalization capability, suggesting potential for structural or training optimization.

To quantitatively evaluate the prediction accuracy of the four models (SSA-BP, SVR, XGB, and BP) for the loess compression deformation coefficient, this study employs four evaluation metrics. Mean Absolute Error (MAE) directly measures the average level of absolute deviation between predicted and true values, reflecting the general accuracy of predictions. Mean Absolute Percentage Error (MAPE) eliminates dimensional effects by characterizing the average percentage magnitude of prediction errors relative to true values, making it more suitable for evaluating relative performance across differently scaled data. Root Mean Square Error (RMSE) assigns higher weight to larger errors through squared terms, effectively representing the overall dispersion of predictions and their sensitivity to outliers. Coefficient of Determination (R^2^) evaluates the proportion of the target variable’s total variability explained by the model. Values closer to 1 indicate higher goodness-of-fit and stronger capability to capture underlying data patterns.

The calculation formulas for these four metrics are given in Eqs. (4) to (7).


$$\begin{array}{c} MAE=\frac{\text{1}}{n}\sum {\mathrm{\backslash nolimits}}_{i=\text{1}}^{n}\left|y_{i}-{\hat{y}}_{i}\right| \end{array}$$ (4)

$$\begin{array}{c} RMSE=\sqrt{\frac{\text{1}}{n}\sum {\mathrm{\backslash nolimits}}_{i=\text{1}}^{n}{\left(y_{i}-{\hat{y}}_{i}\right)}^{\text{2}}} \end{array}$$ (5)

$$\begin{array}{c} MAPE=\frac{\text{1}}{n}\sum {\mathrm{\backslash nolimits}}_{i=\text{1}}^{n}\left|\frac{y_{i}-{\hat{y}}_{i}}{y_{i}}\right|\times \text{1}\text{0}\text{0}\% \end{array}$$ (6)

$$R^{\text{2}}=\text{1}-\frac{\sum_{i=\text{1}}^{n}{\left(y_{i}-{\hat{y}}_{i}\right)}^{\text{2}}}{\sum_{i=\text{1}}^{n}{\left(y_{i}-{\overset{―}{y}}_{i}\right)}^{\text{2}}}$$ (7)

where, $y_{i}$ represents the actual values, ${\hat{y}}_{i}$ represents the predicted values, $\overset{―}{y}$ is the mean of the actual values, *n* is the total number of samples.

**Fig 20:**
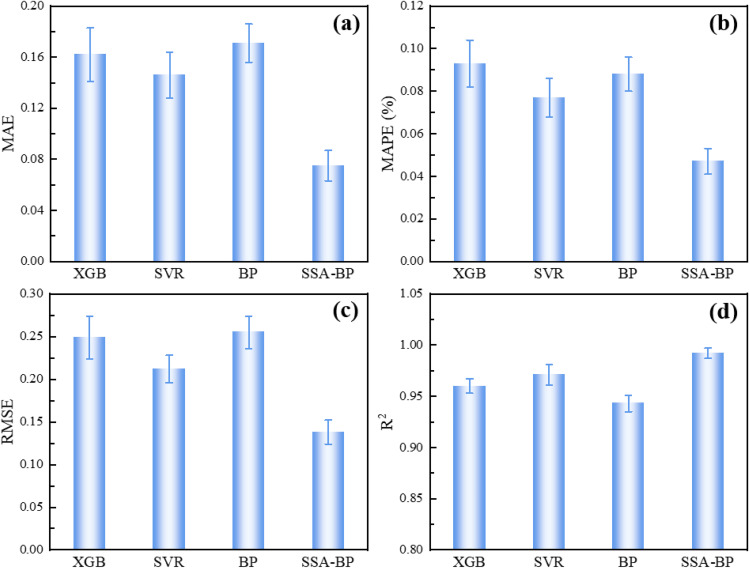
Analysis of statistical indicators for the four models: (a) MAE; (b) MAPE; (c) RMSE; (d) R^2^.

To ensure a statistically robust evaluation and mitigate the bias inherent in a single test split, the final performance of all models was assessed using 10-fold cross-validation. The performance metrics of all models are shown in Fig 20.

The SSA-BP model demonstrates superior performance across all evaluation metrics. It achieves the lowest mean error, with an MAE of 0.075%, MAPE of 0.047, and RMSE of 0.138%, coupled with the highest mean coefficient of determination (R^2^) of 0.992. Crucially, the SSA-BP model also exhibits the smallest standard deviations for these metrics, indicating highly stable and consistent predictions across different data partitions. This robustness is a direct benefit of the Sparrow Search Algorithm’s global optimization of the network’s initial parameters, which effectively prevents the model from converging to suboptimal local minima—a common pitfall of the standard BP network.

The SVR model secures the second-best position, showing balanced performance with moderate error metrics and low standard deviations, which can be attributed to its strong regularization and kernel-based mapping. The XGB model, while achieving a respectable R^2^, shows higher mean errors and greater variability compared to SVR and SSA-BP, suggesting a slight overfitting tendency on this dataset. The traditional BP neural network ranks last in terms of both accuracy and stability, as reflected by its highest mean errors, lowest R^2^, and notable variability. This performance confirms its inherent limitations, including high sensitivity to initial random weights and a propensity to get trapped in local optima during training.

The hierarchy of model performance, quantitatively established through cross-validation, is unequivocally: SSA-BP > SVR > XGB > BP. The SSA-BP model, by synergizing global heuristic search with local gradient optimization, delivers a significant leap in both predictive accuracy and robustness for estimating the loess compression deformation coefficient.

### 5.2. SHAP interpretable analysis

SHAP is an interpretability analysis method grounded in game theory. Its core principle treats each feature as a “player” in a cooperative game, quantifying the global importance and local influence of features by calculating their marginal contribution (Shapley value) to the model’s predictive output across different feature combinations.

**Fig 21:**
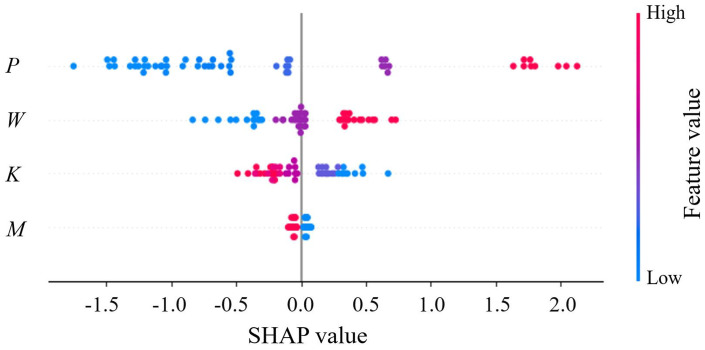
SHAP summary plot.

**Fig 22:**
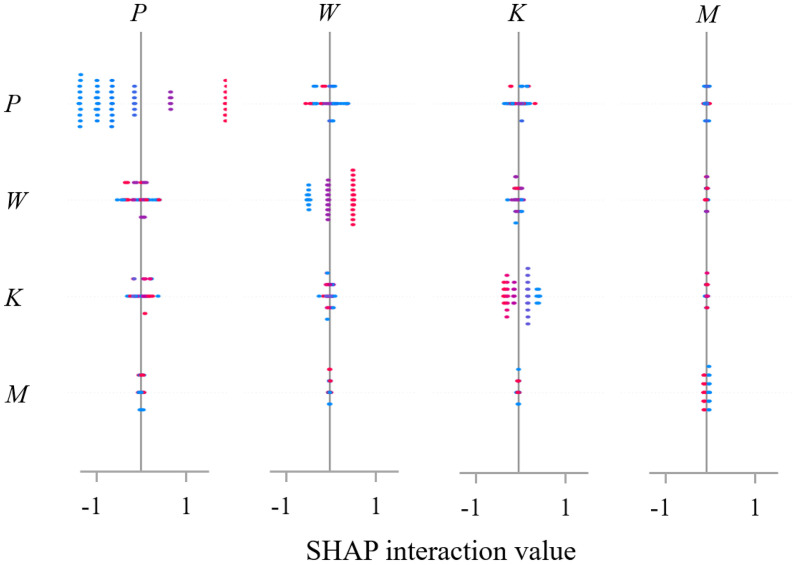
Interaction plot.

In this study, SHAP Summary Plots and Interaction Plots were generated based on the SSA-BP model to quantitatively illustrate the importance ranking and interaction effects of input features, as shown in Figs 21 and 22.

The global feature importance, as depicted by the mean in Fig 21, is quantitatively ranked as: vertical pressure > water content > compaction > molding method. This ranking is visible in the plot, where the feature Vertical pressure occupies the top position and exhibits the widest distribution of SHAP values, indicating its strongest and most variable impact on the model’s output.

Vertical Pressure: The SHAP summary plot for vertical pressure reveals a clear, monotonic positive correlation. As evidenced by the color gradient from blue to red on the x-axis, low SHAP values (blue) are clustered at lower pressure values (left side), while high SHAP values (red/yellow) are concentrated at higher pressure values (right side). Vertical pressure serves as the primary energy input that directly overcomes interparticle resistance. Its dominant role stems from elevated vertical pressure intensifies stress concentration at particle contacts, promoting particle crushing and pore collapse, thereby directly increasing deformation sensitivity.

Water Content: In the summary plot, the points for Water content show a distinct color gradient from blue (low) to red (high) as one moves from left (negative SHAP) to right (positive SHAP) on the x-axis. This demonstrates that high water content values (red points) are predominantly associated with positive SHAP values, meaning the model systematically predicts higher deformation when water content increases. The physical interpretation is the lubricating effect of water, which reduces inter-particle friction.

Compaction: The feature Compaction shows an inverse relationship. In Fig 21, high compaction values (red points) are clustered in the region of negative SHAP values (left side), explicitly showing that increased compaction contributes to a lower predicted deformation coefficient. This visually confirms that denser packing enhances deformation resistance.

Beyond individual effects, the analysis revealed critical nonlinear interactions between features. The vertical pressure-water content interaction was particularly noteworthy: high vertical pressure was found to dramatically amplify the deformation potential induced by increased water content. This synergistic effect can be attributed to the obstruction of water migration under high confining pressure, leading to a surge in local pore water pressure that markedly weakens inter-particle contacts. This finding directly underscores the critical importance of effective drainage systems in loess subgrades subjected to heavy traffic loads, and warrants special attention to stability assessment during rainfall events when water content increases. The analysis confirms that the combined effect of water infiltration and structural loads can lead to substantially higher deformations than would be predicted by considering these factors in isolation, providing a scientific basis for more conservative safety factors in saturation-prone areas. Conversely, the interaction between vertical pressure and compaction exhibited a compensatory nature; higher compaction degrees were shown to mitigate the deformation caused by increased vertical pressure, underscoring the practical value of compaction control in engineering. These interaction patterns, vividly illustrated in the SHAP dependence plots, fundamentally demonstrate that loess deformation is governed by a “load-water” coupling mechanism, moving beyond simplistic univariate analysis to provide a mechanistic anchor for settlement prediction under multifactorial conditions.

## 6. Conclusions

This study experimentally reveals the influence mechanisms of compaction, water content, vertical pressure, and compaction methods on the loess compression deformation coefficient, and constructs a high-precision prediction model based on machine learning algorithms. The main conclusions are as follows:

(1) Loess compression deformation is governed by multi-factor synergy. Increased compaction significantly suppresses deformation by reducing void ratio, while elevated vertical pressure induces nonlinear compression. Higher water content exacerbates deformation through water lubrication that weakens inter-particle friction. Furthermore, vibration compaction reduces the compression deformation coefficient compared to static compaction, owing to dynamic loading that promotes directional particle rearrangement into a homogeneous dense structure, with this advantage amplifying progressively at higher compaction.
(2) The SSA-BP model achieves the highest prediction accuracy (testing set R^2^ = 0.987, MAE = 0.084%) by globally optimizing neural network initial weights and thresholds via the Sparrow Search Algorithm. SVR demonstrates secondary generalization capability through kernel space mapping and regularization constraints. XGB exhibits strong training performance but noticeable test-set overfitting. The traditional BP neural network yields the lowest accuracy due to local minima limitations.
(3) By combining the sparrow search algorithm with the BP neural network, the SSA-BP model overcomes the limitations of local minima and slow convergence speed through global weight threshold optimization, which greatly improves the prediction accuracy and robustness of loess compression features. As a result, the SSA-BP model shows the best performance in predicting the compression deformation coefficients of loess subgrade.
(4) SHAP analysis reveals that the importance of the features were ranked in the order of vertical pressure, water content, compaction and molding method on loess compression deformation, with vertical pressure serving as the globally dominant feature. Significant interaction effects exist between vertical pressure-water content and vertical pressure-compaction regarding their influence on the compression deformation coefficient of loess subgrade. This fundamentally demonstrates that loess deformation behavior is governed by the coupling effect of “load and water” interactions.
(5) This study provides a reliable data-driven tool for predicting loess compression deformation, offering valuable support for loess subgrade design and stability assessment. However, the models were developed using a limited laboratory dataset (n = 180) from a single loess source, and their performance under field conditions or with different loess compositions requires further verification. Future research should focus on expanding dataset diversity and size, validating predictions with field monitoring data, incorporating additional factors, and integrating the data-driven approach with physics-based models to enhance both predictive accuracy and physical interpretability.

## Supporting information

S1 Table Complete database. (DOCX)
