# Supplementary material for: Influence mechanism exploration and machine learning prediction of loess compression deformation coefficient under multi-factor coupling effects
Source: PLoS One. 2026 Jan 9;21(1):e0338428. doi: 10.1371/journal.pone.0338428 (PMC12788692; doi:10.1371/journal.pone.0338428)
Supplement: S1 Table — (DOCX) [file pone.0338428.s001.docx]

| Compaction method | Vertical pressure | Water content | Compaction | Deformation coefficient |
| --- | --- | --- | --- | --- |
| hydrostatic method | 50 | 12 | 0.9 | 0.45 |
| hydrostatic method | 50 | 12 | 0.93 | 0.35 |
| hydrostatic method | 50 | 12 | 0.96 | 0.3 |
| hydrostatic method | 50 | 12 | 0.98 | 0.26 |
| hydrostatic method | 50 | 12 | 1 | 0.23 |
| hydrostatic method | 50 | 14 | 0.9 | 0.65 |
| hydrostatic method | 50 | 14 | 0.93 | 0.52 |
| hydrostatic method | 50 | 14 | 0.96 | 0.48 |
| hydrostatic method | 50 | 14 | 0.98 | 0.43 |
| hydrostatic method | 50 | 14 | 1 | 0.39 |
| hydrostatic method | 50 | 16 | 0.9 | 0.79 |
| hydrostatic method | 50 | 16 | 0.93 | 0.66 |
| hydrostatic method | 50 | 16 | 0.96 | 0.54 |
| hydrostatic method | 50 | 16 | 0.98 | 0.48 |
| hydrostatic method | 50 | 16 | 1 | 0.5 |
| vibration method | 50 | 12 | 0.9 | 0.45 |
| vibration method | 50 | 12 | 0.93 | 0.34 |
| vibration method | 50 | 12 | 0.96 | 0.31 |
| vibration method | 50 | 12 | 0.98 | 0.24 |
| vibration method | 50 | 12 | 1 | 0.21 |
| vibration method | 50 | 14 | 0.9 | 0.62 |
| vibration method | 50 | 14 | 0.93 | 0.52 |
| vibration method | 50 | 14 | 0.96 | 0.44 |
| vibration method | 50 | 14 | 0.98 | 0.41 |
| vibration method | 50 | 14 | 1 | 0.39 |
| vibration method | 50 | 16 | 0.9 | 0.75 |
| vibration method | 50 | 16 | 0.93 | 0.65 |
| vibration method | 50 | 16 | 0.96 | 0.53 |
| vibration method | 50 | 16 | 0.98 | 0.46 |
| vibration method | 50 | 16 | 1 | 0.49 |
| hydrostatic method | 100 | 12 | 0.9 | 0.8 |
| hydrostatic method | 100 | 12 | 0.93 | 0.71 |
| hydrostatic method | 100 | 12 | 0.96 | 0.64 |
| hydrostatic method | 100 | 12 | 0.98 | 0.62 |
| hydrostatic method | 100 | 12 | 1 | 0.54 |
| hydrostatic method | 100 | 14 | 0.9 | 1.11 |
| hydrostatic method | 100 | 14 | 0.93 | 0.95 |
| hydrostatic method | 100 | 14 | 0.96 | 0.89 |
| hydrostatic method | 100 | 14 | 0.98 | 0.82 |
| hydrostatic method | 100 | 14 | 1 | 0.75 |
| hydrostatic method | 100 | 16 | 0.9 | 1.31 |
| hydrostatic method | 100 | 16 | 0.93 | 1.12 |
| hydrostatic method | 100 | 16 | 0.96 | 0.98 |
| hydrostatic method | 100 | 16 | 0.98 | 0.92 |
| hydrostatic method | 100 | 16 | 1 | 0.91 |
| vibration method | 100 | 12 | 0.9 | 0.79 |
| vibration method | 100 | 12 | 0.93 | 0.68 |
| vibration method | 100 | 12 | 0.96 | 0.63 |
| vibration method | 100 | 12 | 0.98 | 0.57 |
| vibration method | 100 | 12 | 1 | 0.51 |
| vibration method | 100 | 14 | 0.9 | 1.1 |
| vibration method | 100 | 14 | 0.93 | 0.92 |
| vibration method | 100 | 14 | 0.96 | 0.84 |
| vibration method | 100 | 14 | 0.98 | 0.76 |
| vibration method | 100 | 14 | 1 | 0.69 |
| vibration method | 100 | 16 | 0.9 | 1.29 |
| vibration method | 100 | 16 | 0.93 | 1.09 |
| vibration method | 100 | 16 | 0.96 | 0.95 |
| vibration method | 100 | 16 | 0.98 | 0.88 |
| vibration method | 100 | 16 | 1 | 0.88 |
| hydrostatic method | 200 | 12 | 0.9 | 1.1 |
| hydrostatic method | 200 | 12 | 0.93 | 0.97 |
| hydrostatic method | 200 | 12 | 0.96 | 0.88 |
| hydrostatic method | 200 | 12 | 0.98 | 0.83 |
| hydrostatic method | 200 | 12 | 1 | 0.75 |
| hydrostatic method | 200 | 14 | 0.9 | 1.55 |
| hydrostatic method | 200 | 14 | 0.93 | 1.3 |
| hydrostatic method | 200 | 14 | 0.96 | 1.18 |
| hydrostatic method | 200 | 14 | 0.98 | 1.1 |
| hydrostatic method | 200 | 14 | 1 | 1.01 |
| hydrostatic method | 200 | 16 | 0.9 | 1.91 |
| hydrostatic method | 200 | 16 | 0.93 | 1.65 |
| hydrostatic method | 200 | 16 | 0.96 | 1.44 |
| hydrostatic method | 200 | 16 | 0.98 | 1.35 |
| hydrostatic method | 200 | 16 | 1 | 1.33 |
| vibration method | 200 | 12 | 0.9 | 1.07 |
| vibration method | 200 | 12 | 0.93 | 0.93 |
| vibration method | 200 | 12 | 0.96 | 0.85 |
| vibration method | 200 | 12 | 0.98 | 0.76 |
| vibration method | 200 | 12 | 1 | 0.69 |
| vibration method | 200 | 14 | 0.9 | 1.51 |
| vibration method | 200 | 14 | 0.93 | 1.24 |
| vibration method | 200 | 14 | 0.96 | 1.11 |
| vibration method | 200 | 14 | 0.98 | 1 |
| vibration method | 200 | 14 | 1 | 0.92 |
| vibration method | 200 | 16 | 0.9 | 1.89 |
| vibration method | 200 | 16 | 0.93 | 1.59 |
| vibration method | 200 | 16 | 0.96 | 1.37 |
| vibration method | 200 | 16 | 0.98 | 1.27 |
| vibration method | 200 | 16 | 1 | 1.26 |
| hydrostatic method | 400 | 12 | 0.9 | 1.56 |
| hydrostatic method | 400 | 12 | 0.93 | 1.43 |
| hydrostatic method | 400 | 12 | 0.96 | 1.3 |
| hydrostatic method | 400 | 12 | 0.98 | 1.21 |
| hydrostatic method | 400 | 12 | 1 | 1.1 |
| hydrostatic method | 400 | 14 | 0.9 | 2.15 |
| hydrostatic method | 400 | 14 | 0.93 | 1.93 |
| hydrostatic method | 400 | 14 | 0.96 | 1.71 |
| hydrostatic method | 400 | 14 | 0.98 | 1.56 |
| hydrostatic method | 400 | 14 | 1 | 1.43 |
| hydrostatic method | 400 | 16 | 0.9 | 2.67 |
| hydrostatic method | 400 | 16 | 0.93 | 2.37 |
| hydrostatic method | 400 | 16 | 0.96 | 2.18 |
| hydrostatic method | 400 | 16 | 0.98 | 2.04 |
| hydrostatic method | 400 | 16 | 1 | 2.04 |
| vibration method | 400 | 12 | 0.9 | 1.5 |
| vibration method | 400 | 12 | 0.93 | 1.33 |
| vibration method | 400 | 12 | 0.96 | 1.22 |
| vibration method | 400 | 12 | 0.98 | 1.09 |
| vibration method | 400 | 12 | 1 | 1.01 |
| vibration method | 400 | 14 | 0.9 | 2.08 |
| vibration method | 400 | 14 | 0.93 | 1.78 |
| vibration method | 400 | 14 | 0.96 | 1.63 |
| vibration method | 400 | 14 | 0.98 | 1.4 |
| vibration method | 400 | 14 | 1 | 1.29 |
| vibration method | 400 | 16 | 0.9 | 2.59 |
| vibration method | 400 | 16 | 0.93 | 2.25 |
| vibration method | 400 | 16 | 0.96 | 2.05 |
| vibration method | 400 | 16 | 0.98 | 1.92 |
| vibration method | 400 | 16 | 1 | 1.88 |
| hydrostatic method | 800 | 12 | 0.9 | 2.31 |
| hydrostatic method | 800 | 12 | 0.93 | 2.17 |
| hydrostatic method | 800 | 12 | 0.96 | 1.93 |
| hydrostatic method | 800 | 12 | 0.98 | 1.85 |
| hydrostatic method | 800 | 12 | 1 | 1.71 |
| hydrostatic method | 800 | 14 | 0.9 | 3.09 |
| hydrostatic method | 800 | 14 | 0.93 | 2.74 |
| hydrostatic method | 800 | 14 | 0.96 | 2.43 |
| hydrostatic method | 800 | 14 | 0.98 | 2.13 |
| hydrostatic method | 800 | 14 | 1 | 1.98 |
| hydrostatic method | 800 | 16 | 0.9 | 3.66 |
| hydrostatic method | 800 | 16 | 0.93 | 3.36 |
| hydrostatic method | 800 | 16 | 0.96 | 3.17 |
| hydrostatic method | 800 | 16 | 0.98 | 2.89 |
| hydrostatic method | 800 | 16 | 1 | 2.86 |
| vibration method | 800 | 12 | 0.9 | 2.24 |
| vibration method | 800 | 12 | 0.93 | 2.06 |
| vibration method | 800 | 12 | 0.96 | 1.82 |
| vibration method | 800 | 12 | 0.98 | 1.68 |
| vibration method | 800 | 12 | 1 | 1.53 |
| vibration method | 800 | 14 | 0.9 | 2.97 |
| vibration method | 800 | 14 | 0.93 | 2.69 |
| vibration method | 800 | 14 | 0.96 | 2.35 |
| vibration method | 800 | 14 | 0.98 | 1.99 |
| vibration method | 800 | 14 | 1 | 1.87 |
| vibration method | 800 | 16 | 0.9 | 3.55 |
| vibration method | 800 | 16 | 0.93 | 3.13 |
| vibration method | 800 | 16 | 0.96 | 2.92 |
| vibration method | 800 | 16 | 0.98 | 2.68 |
| vibration method | 800 | 16 | 1 | 2.68 |
| hydrostatic method | 1600 | 12 | 0.9 | 3.32 |
| hydrostatic method | 1600 | 12 | 0.93 | 3.04 |
| hydrostatic method | 1600 | 12 | 0.96 | 2.85 |
| hydrostatic method | 1600 | 12 | 0.98 | 2.74 |
| hydrostatic method | 1600 | 12 | 1 | 2.56 |
| hydrostatic method | 1600 | 14 | 0.9 | 4.7 |
| hydrostatic method | 1600 | 14 | 0.93 | 4.05 |
| hydrostatic method | 1600 | 14 | 0.96 | 3.56 |
| hydrostatic method | 1600 | 14 | 0.98 | 3.15 |
| hydrostatic method | 1600 | 14 | 1 | 2.98 |
| hydrostatic method | 1600 | 16 | 0.9 | 5.71 |
| hydrostatic method | 1600 | 16 | 0.93 | 5.16 |
| hydrostatic method | 1600 | 16 | 0.96 | 4.46 |
| hydrostatic method | 1600 | 16 | 0.98 | 4.29 |
| hydrostatic method | 1600 | 16 | 1 | 4.24 |
| vibration method | 1600 | 12 | 0.9 | 3.18 |
| vibration method | 1600 | 12 | 0.93 | 2.95 |
| vibration method | 1600 | 12 | 0.96 | 2.74 |
| vibration method | 1600 | 12 | 0.98 | 2.6 |
| vibration method | 1600 | 12 | 1 | 2.47 |
| vibration method | 1600 | 14 | 0.9 | 4.5 |
| vibration method | 1600 | 14 | 0.93 | 3.83 |
| vibration method | 1600 | 14 | 0.96 | 3.32 |
| vibration method | 1600 | 14 | 0.98 | 2.89 |
| vibration method | 1600 | 14 | 1 | 2.76 |
| vibration method | 1600 | 16 | 0.9 | 5.51 |
| vibration method | 1600 | 16 | 0.93 | 4.83 |
| vibration method | 1600 | 16 | 0.96 | 4.25 |
| vibration method | 1600 | 16 | 0.98 | 3.96 |
| vibration method | 1600 | 16 | 1 | 3.92 |
